# Saturated fatty acid- and/or monounsaturated fatty acid-containing-phosphatidic acids selectively interact with and activate phosphoglycerate mutase 1

**DOI:** 10.1016/j.bbrep.2025.102285

**Published:** 2025-09-29

**Authors:** Kamila Dilimulati, Naoto Yachida, Fumi Hoshino, Fumio Sakane

**Affiliations:** Department of Chemistry, Graduate School of Science, Chiba University, 1-33 Yayoi-cho, Inage-ku, Chiba, 263-8522, Japan

**Keywords:** Phosphatidic acid, Phosphoglycerate mutase 1, Saturated fatty acid, Monounsaturated fatty acid, Diacylglycerol kinase, Secondary structure

## Abstract

This study aimed to identify the target proteins of 16:0/16:0-phosphatidic acid (PA), which is produced by diacylglycerol kinases (DGKs) α, δ, and ζ. We identified phosphoglycerate mutase 1 (PGAM1), a key glycolytic enzyme that catalyzes the conversion of 3-phosphoglycerate to 2-phosphoglycerate, as a PA-binding protein with a stronger affinity for PA than for other phospholipids, including phosphatidylinositol, phosphatidylinositol 4-monophosphate, phosphatidylinositol 4,5-bisphosphate, cardiolipin, phosphatidylserine, phosphatidylglycerol, phosphatidylcholine, and sphingomyelin. PGAM1 preferentially binds to saturated fatty acid (SFA)- and/or monounsaturated fatty acid (MUFA)-containing PAs, such as 16:0/16:0-, 16:0/18:1-, 18:0/18:0-, 18:0/18:1-, and 18:1/18:1-PA, compared to polyunsaturated fatty acid-containing PAs, such as 18:0/20:4- and 18:0/22:6-PA. Notably, 16:0/16:0- and 16:0/18:1-PA altered the secondary conformation of PGAM1 and substantially enhanced its activity. Interestingly, PGAM1 interacted with DGKδ and ζ, but not with DGKα. These findings indicate that SFA- and/or MUFA-containing-PAs selectively interact with PGAM1, a promising therapeutic target for cancer, type 2 diabetes mellitus, and senescence, to regulate its activity.

## Highlights

• Phosphoglycerate mutase 1 (PGAM1) is a key glycolytic enzyme.
• PGAM1 preferentially binds to phosphatidic acid (PA) compared to other phospholipids.
• PGAM1 preferentially binds to 16:0/16:0-, 16:0/18:1-, 18:0/18:0-, 18:0/18:1-, and 18:1/18:1-PA.
• 16:0/16:0- and 16:0/18:1-PA altered the secondary conformation of PGAM1 and enhanced its activity.
• PGAM1 interacted with diacylglycerol kinase (DGK) δ and ζ, but not with DGKα.

## 1 Introduction

Phosphatidic acid (PA) is a crucial signaling lipid that interacts with numerous proteins, with over 70 PA-binding proteins identified to date [[1], [2], [3], [4], [5]]. PA-binding proteins include atypical protein kinase C (PKC) ζ [6], novel PKC isoforms (PKCδ [7] and ε [8,9]), C-Raf [[10], [11], [12]], cAMP phosphodiesterase 4A1 [13,14], OverProducer of Inositol 1 [14,15], sporulation-specific protein 20 [14,16,17], phosphatidylinositol (PI) 4,5-bisphosphate (PI-4,5-P_2_)-specific phospholipase C (PLC) β1 [18], α-synuclein [19,20], creatine kinase muscle type (CKM) [21], Praja-1 [22], l-lactate dehydrogenase A [23], synaptojanin-1 [24], and clathrin coat assembly protein AP180 [25].

Diacylglycerol kinase (DGK) phosphorylates diacylglycerol (DG) to produce PA [[26], [27], [28], [29]] and participates in various pathological and physiological processes [30,31]. DGKα [32,33], which contains tandem Ca^2+^-binding EF-hand motif domains, is activated by Ca^2+^ [32,[34], [35], [36], [37], [38]]. In T lymphocytes, DGKα facilitates an immune non-responsive (non-proliferative) state known as anergy [[39], [40], [41]]. Conversely, DGKα promotes proliferation and attenuates apoptosis in hepatocellular carcinoma cells [42] and melanoma [[43], [44], [45]], while enhancing epithelial-mesenchymal transition (metastasis) in glioblastoma cells [46]. In skeletal muscle, DGKδ is a critical enzyme in the pathogenesis of type 2 diabetes mellitus [47], and in the brain, it regulates the serotonergic system [22,48]. DGKζ downregulates T cell responses [49] and plays a crucial role in neuronal cells, maintaining spinal density [50], and influencing hippocampal long-term potentiation/depression [51]. Our recent studies have shown that DGKα preferentially generates saturated fatty acid (SFA)- and/or monounsaturated fatty acid (MUFA)-containing PAs, such as 16:0/16:0-, 16:0/16:1-, and 16:0/18:0-PA in AKI melanoma cells [44], whereas DGKδ selectively produces SFA- and/or MUFA-containing PAs, such as 14:0/16:0-, 14:0/16:1-, 16:0/16:0-, 16:0/16:1-, 16:0/18:0-, and 16:0/18:1-PAs in myoblasts [52]. Additionally, DGKζ selectively generates SFA-containing PAs such as 14:0/16:0-, 16:0/16:0-, and 16:0/18:0-PAs during neuronal cell differentiation [53].

Recently, we searched for target proteins of 16:0/16:0-PA in myoblasts [21,23] and melanoma cells [54], and identified CKM [21] and heat shock protein 27 (HSP27) [54] as 16:0/16:0-PA-binding proteins. We identified phosphoglycerate mutase (PGAM) 2 (Accession No. O70250) and PGAM1 (Accession No. P18669) as novel PA-binding proteins in myoblasts and melanoma cells, respectively. PGAM, a key glycolytic enzyme, catalyzes the conversion of 3-phosphoglycerate to 2-phosphoglycerate [55]. PGAM1 and 2 share high sequence homology (80.7 % identity, 90.2 % similarity). Although PGAM2 is primarily expressed in the skeletal muscle and testes, PGAM1 is ubiquitously detected in various tissues, including the skeletal muscles and testes (https://www.proteinatlas.org). PGAM1 plays an essential role in tumor growth [56,57] and is a promising target for cancer therapy [[58], [59], [60]]. Moreover, PGAM1 is considered as an attractive target for type 2 diabetes [61] and senescence [62,63].

In this study, we characterized PGAM1 as a PA-binding protein and found that it preferentially binds to PA compared to other phospholipids, including PI, PI 4-monophosphate, PI 4,5-bisphosphate, phosphatidylserine (PS), phosphatidylglycerol (PG), cardiolipin (CL), and phosphatidylcholine (PC). Our results showed that PGAM1 interacted more strongly with SFA- and/or MUFA-containing PAs, such as 16:0/16:0-, 16:0/18:1-, 18:0/18:0-, 18:0/18:1-, and 18:1/18:1-PA, than with polyunsaturated fatty acid (PUFA)-containing PAs, such as 18:0/22:6- and 18:0/20:4-PA. Notably, 16:0/16:0-, 16:0/18:1-, 18:0/18:0-, and 18:0/18:1-PA altered the secondary conformation of PGAM1, and 16:0/16:0- and 16:0/18:1-PA significantly enhanced its activity. Intriguingly, PGAM1 interacted with DGKδ and ζ, but not with DGKα.

## 2 Materials and methods

### 2.1 Materials

l-α-PC from egg yolk, 1,2-dipalmitoyl-sn-glycero-3-phosphoserine (16:0/16:0-PS), 1,2-dipalmitoyl-sn-glycero-3-phosphate (16:0/16:0-PA), 1-palmitoyl-2-oleoyl-sn-glycero-3-phosphate (16:0/18:1-PA), 1,2-dioleoyl-sn-glycero-3-phosphate (18:1/18:1-PA), 1-stearoyl-2-oleoyl-sn-glycero-3-phosphate (18:0/18:1-PA), 1,2-distearoyl-sn-glycero-3-phosphate (18:0/18:0-PA), 1-stearoyl-2-arachidonoyl-sn-glycero-3-phosphate (18:0/20:4-PA), and 1-stearoyl-2-docosahexaenoyl-sn-glycero-3-phosphate (18:0/22:6-PA) were bought from Avanti Polar Lipids (Alabaster, AL, USA). Cholesterol was obtained from Wako Pure Chemical Industries (Tokyo, Japan). Membrane Lipid Strips were bought from Echelon Biosciences (Salt Lake City, UT, USA).

### 2.2 Protein expression and purification of PGAM1

PGAM1 cDNA (Accession number: NM_002629) was amplified from human AKI cell cDNA using the following primers: forward 5′- GGTGGTGGATCCATGGCCGCCTACAAACTG-3′ and reverse 5′- GGTGGTCTCGAGTCACTTCTTGGCCTTGCC-3′. The amplified product was ligated with the pET-28a vector (Novagen-Merck, Darmstadt, Germany) and transfected into Rosetta 2 (DE3) *Escherichia coli* cells (Novagen-Merck). The 6 × His-tagged PGAM1 protein was expressed and purifed using nickel-nitrilotriacetic acid (Ni-NTA) agarose (Qiagen, Hilden, Germany), as previously described [35,64].

### 2.3 Western blotting

Western blotting was performed as described previously [65]. Purified proteins and cell lysates were separated using sodium dodecyl sulfate (SDS)–polyacrylamide gel electrophoresis (PAGE) and transferred to a polyvinylidene fluoride membrane (Pall Corporation, Tokyo, Japan). The membrane was blocked with 5 % (w/w) skim milk and incubated for 1 h with primary antibodies diluted in 5 % (w/w) skim milk. The following primary antibodies were used: anti-DGKα (Proteintech, Rosemont, IL, USA; 11547-1-AP; 1:1000), anti-DGKδ ([66]; 1:1000), anti-DGKζ (LSBio, Newark, CA, USA; LS-C289672; 1:1000), and anti-6 × His (Fujifilm Wako, Tokyo, Japan; 010–21861; 1:1000). After washing, the membranes were incubated with horseradish peroxidase-conjugated anti-rabbit or anti-mouse IgG secondary antibodies (Cell Signaling Technology, Danvers, MA, USA). Protein bands were visualized using Pierce ECL Western Blotting Substrate (Thermo Fisher Scientific, Waltham, MA, USA).

### 2.4 Preparation of liposomes

The following lipid mixtures were used to determine the properties of the PGAM1 protein: control liposomes [cholesterol (30 mol%) and PC Mix (from egg yolk) (70 mol%)], PS liposomes [cholesterol (30 mol%), PC Mix (from egg yolk) (50 mol%), and 16:0/16:0-PS (20 mol%)], and PA liposomes [(cholesterol (30 mol%), PC Mix (from egg yolk) (50 mol%), and each PA species (20 mol%)]. For the lipid-binding assay, the dried lipid mixture was hydrated in HEPES buffer at 95 °C for 45 min, with vortexing for 1 min after every 15 min. The liposomes were then subjected to five freeze-thaw cycles (−196 °C for 3 min, 95 °C for 3 min). Liposome formation was induced by sonication at 90 °C using a Branson Sonifier 450. Because lipids form bilayers, half of the actual concentration was considered [67].

### 2.5 Liposome-binding assay

Purified 6 × His-tagged PGAM1 protein (0.5 μM final concentration) was dissolved in HEPES buffer and incubated with PA-containing or control liposomes at 4 °C for 30 min. Samples were ultracentrifuged at 200,000 *g* at 4 °C for 1 h. The resulting precipitate was dissolved in HEPES buffer. Proteins were separated by 15 % SDS–PAGE and stained with Coomassie Brilliant Blue (CBB).

### 2.6 Protein-lipid overlay assay

Various lipids (100 pmol each) were spotted onto nitrocellulose membranes (Lipid Strips; Echelon Biosciences). Membranes were blocked with 2 % (w/w) skim milk in phosphate-buffered saline (PBS, pH 7.4) for 1 h at 4 °C. After blocking, the membranes were incubated with 10 mL of 3 % (w/w) fatty acid (FA)-free bovine serum albumin and 0.1 % Tween 20 in PBS (pH 7.4) containing 6 × His-tagged PGAM1 (40 nM final concentration) for 20 min at 4 °C. Membranes were then incubated with an anti-6 × His antibody (D291-3S, Medical & Biological Laboratories, Nagoya, Japan) for 1 h at 4 °C, followed by incubation with horseradish peroxidase-conjugated anti-mouse IgG antibody (Bethyl Laboratories, Montgomery, TX, USA). Lipid-bound 6 × His-PGAM1 was visualized using Pierce ECL Western Blotting Substrate (Thermo Fisher Scientific, Waltham, MA, USA).

### 2.7 Densitometry

Band and spot signal intensities from liposome-binding and Western blot analyses were quantified using ImageJ software (https://imagej.nih.gov/ij/index.html), as described by Miller (https://lukemiller.org/index.php/2010/11/analyzing-gels-and-western-blots-with-image-j/).

### 2.8 Circular dichroism (CD) spectroscopy

CD spectra were recorded under ambient conditions between 190 nm and 250 nm using a Jasco J-805 spectrometer (Jasco Corporation, Tokyo, Japan). Measurements were taken with a 0.2 mm path length cell, 20 nm/min scan speed, and 1 nm bandwidth. A 16 μM solution of 6 × His-tagged PGAM1 was prepared in 20 mM Tris-HCl buffer (pH 7.4) containing 1 mM dithiothreitol and control or 12.5 μM PA-containing liposomes. Five spectra were averaged, and the spectrum obtained for the buffer was subtracted. Spectral data were analyzed using the K2D algorithm [68] on the DICHROWEB platform (http://dichroweb. cryst.bbk.ac.uk/html/home.shtml) [69].

### 2.9 PGAM activity assay

PGAM activity was measured using a multiple-enzyme coupled assay, as previously described [70]. The reaction mixture (100 μL) contained 50 mM HEPES (pH 7.5), 10 mM MgCl_2_, 1 mM ADP, 0.5 units/mL enolase (Sigma-Aldrich), and 0.5 units/mL recombinant pyruvate kinase M1 (Sigma-Aldrich). Additionally, recombinant PGAM1 (0.1 mg/mL) was added either without or with 12.5 μM PA liposomes. After a 5-min incubation at 25 °C, the reaction was initiated by adding 3-phosphoglycerate to a final concentration of 1 mM and allowed to proceed for 10 min at 25 °C. ATP production was measured using a Kinase-Glo Luminescent Kinase Assay (Promega, Madison, WI, USA).

### 2.10 Protein-protein interaction analysis

HEK293T cells were lysed in Pierce IP Lysis Buffer (Thermo Fisher Scientific) for 15 min on ice. Lysates were cleared by centrifugation at 12,000 *g* for 15 min at 4 °C. Approximately 50 μg of purified 6 × His-tagged PGAM1 was incubated with 30 μL of pre-equilibrated Ni-NTA agarose beads (Qiagen) in lysis buffer, for 60 min at 4 °C with gentle rotation. After washing the beads three times with lysis buffer, 300 μg of HEK293T cell lysate was added and incubated for 2 h at 4 °C with gentle rotation. The beads were then washed five times with wash buffer (20 mM Tris-HCl, pH 7.5, 300 mM NaCl, 0.1 % Tween-20), and the bound proteins were eluted by boiling the beads in 2 × SDS sample buffer at 95 °C for 5 min.

### 2.11 Statistical analysis

Data are represented as the mean ± S.D. and were analyzed using one-way analysis of variance (ANOVA) followed by Tukey's or Dunnett's post-hoc test for multiple comparisons. Statistical analyses were performed using Prism 8 software (GraphPad Software, San Diego, CA, USA). Statistical significance was set at p < 0.05.

## 3 Results

### 3.1 Identification of PGAM1 as a 16:0/16:0-PA-binding protein

**Fig. 1:**
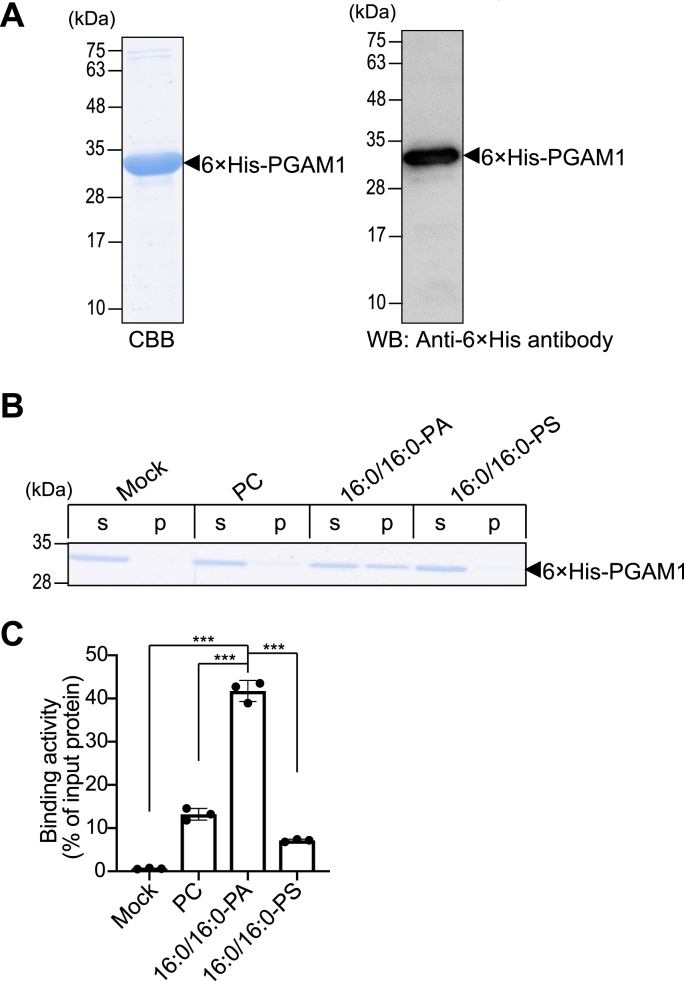
PGAM1 identified as a PA-binding protein (A) The 6 × His-PGAM1 protein expressed in *E. coli* cells was purified, separated by SDS–PAGE (15 % acrylamide), and visualized by CBB staining (*CBB*) and western blotting (*WB*) using an anti-6 × His antibody. A black arrowhead indicates the position of 6 × His-PGAM1 (approximately 32 kDa). (B) Liposome-binding assay of 6 × His-PGAM1 (0.5 μM) with 16:0/16:0-PA, 16:0/16:0-PS, or PC liposomes (200 μM PA or PS). After ultracentrifugation, the proteins were separated by SDS–PAGE (15 % acrylamide) and stained with CBB. Black arrowhead indicates the position of 6 × His-PGAM1 (approximately 32 kDa). (C) Protein amounts in the supernatant (*s*) and precipitate (*p*) were quantified by densitometry using the ImageJ software. Binding activity was calculated as the percentage of precipitate band intensity relative to the total band intensity. Data are presented as the mean ± SD from three independent experiments. ∗∗∗P < 0.005, one-way ANOVA followed by Tukey's post-hoc test.

We previously identified CKM [21] and HSP27 [54] as the target proteins of 16:0/16:0-PA in C2C12 myoblasts and AKI melanoma cells, respectively. During our search for additional 16:0/16:0-PA-binding proteins, we identified PGAM1 (Accession No. P18669) in human melanoma cells and PGAM2 (Accession No. O70250) in mouse skeletal muscle cells as potential candidates (Suppl. Fig. 1). PGAM1 and PGAM2 share high sequence homology (80.7 % identity; 90.2 % similarity). Although PGAM2 is selectively expressed in skeletal muscle and testes, PGAM1 is ubiquitously distributed in many tissues, including the skeletal muscle and testes (https://www.proteinatlas.org). Moreover, PGAM1 plays a crucial role in glycolysis, catalyzing the conversion of 3-phosphoglycerate to 2-phosphoglycerate, and is a promising target in cancer [59,60], type 2 diabetes [61] and senescence [62,63]. Therefore, we focused on PGAM1 for further investigation. Next, we cloned human PGAM1 cDNA (Accession number: NM_002629) from AKI cell mRNAs and ligated it into the pET-28a vector. The 6 × His-tagged PGAM1 protein was produced in *E. coli* and purified by affinity chromatography using Ni-NTA agarose. We confirmed the successful purification of 6 × His-PGAM1, which was detected as a single band with a molecular mass of approximately 32 kDa (Fig. 1A), and was recognized by an anti-6 × His antibody (Fig. 1A).

Next, we examined the binding activity of PGAM1 to 16:0/16:0-PA using a liposome-binding assay [21]. As a background control, only 12 % of PGAM1 was co-sedimented with liposomes containing PC (neutral phospholipid) alone (Fig. 1B and C). Similarly, 16:0/16:0-PS liposomes (200 μM PS), used as an acidic phospholipid control, co-precipitated with 7 % of PGAM1 (Fig. 1B and C). In contrast, approximately 40 % of PGAM1 co-precipitated with 16:0/16:0-PA liposomes (200 μM PA) (Fig. 1B and C), indicating that PGAM1 interacted more strongly with 16:0/16:0-PA liposomes than with 16:0/16:0-PS (acidic phospholipid control) or PC liposomes (background control).

### 3.2 PGAM1 exhibits strongest binding affinity for PA among various lipids

**Fig. 2:**
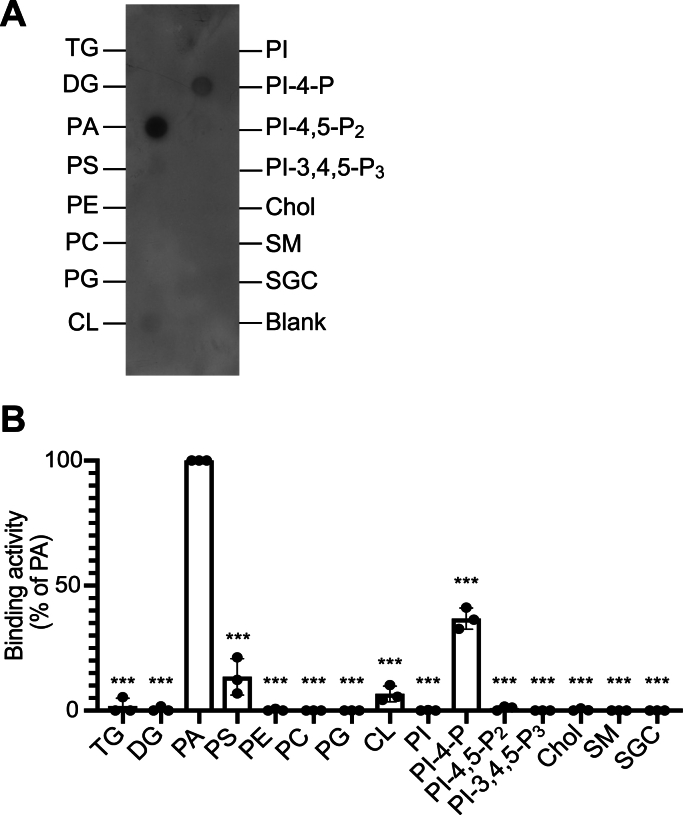
Binding activity of 6 × His-PGAM1 to various lipids (A) Lipid overlay assay of 6 × His-PGAM1 using equimolar amounts (100 pmol) of different lipids (TG, DG, PA, PS, PE, PC, PG, CL, PI, PI-4-P, PI-4,5-P_2_, PI-3,4,5-P_3_, Chol, SM, and SGC) spotted on nitrocellulose membranes (Lipid Strips™, Echelon Biosciences). The acyl chain(s) of these glycerolipids and sphingolipids are C16:0. Membranes were incubated with purified 6 × His-PGAM1 (40 nM). Lipid-bound proteins were detected using an anti-6 × His antibody. Data are representative of three independent experiments. (B) Quantification of spot intensities using the ImageJ software. The binding activity (spot intensity) of PGAM1 to PA was set as 100 %. Data are presented as the mean ± SD from three independent experiments. ∗∗∗P < 0.005 versus PA, one-way ANOVA followed by Tukey's post-hoc test (versus PA).

To further investigate the lipid-binding selectivity of PGAM1, we conducted a lipid overlay assay using a nitrocellulose membrane spotted with diverse lipids containing 16:0 fatty acid chains. PGAM1 demonstrated a strong interaction with 16:0/16:0-PA (Fig. 2A and B). Although other acidic lipids, including PI-4-monophosphate (PI-4-P), PS, and CL, were also associated with PGAM1, their binding intensities were significantly lower than that of PA (Fig. 2A and B). PGAM1 did not bind to other acidic lipids, such as PI, PI-4,5-P_2_, PI-3,4,5-trisphosphate (PI-3,4,5-P_3_), PG and 3-sulfogalactosylceramide (SGC) (Fig. 2A and B). Similarly, neutral lipids, including triacylglycerol (TG), DG, PC, phosphatidylethanolamine (PE), cholesterol (Chol), and sphingomyelin (SM), failed to interact with PGAM1 (Fig. 2A and B). Therefore, these results indicate that PGAM1 selectively and most strongly interacts with PA.

### 3.3 PGAM1 exhibits moderate binding affinity for 16:0/16:0-, 16:0/18:1-, 18:0/18:0-, 18:0/18:1- and 18:1/18:1-PA species

**Fig. 3:**
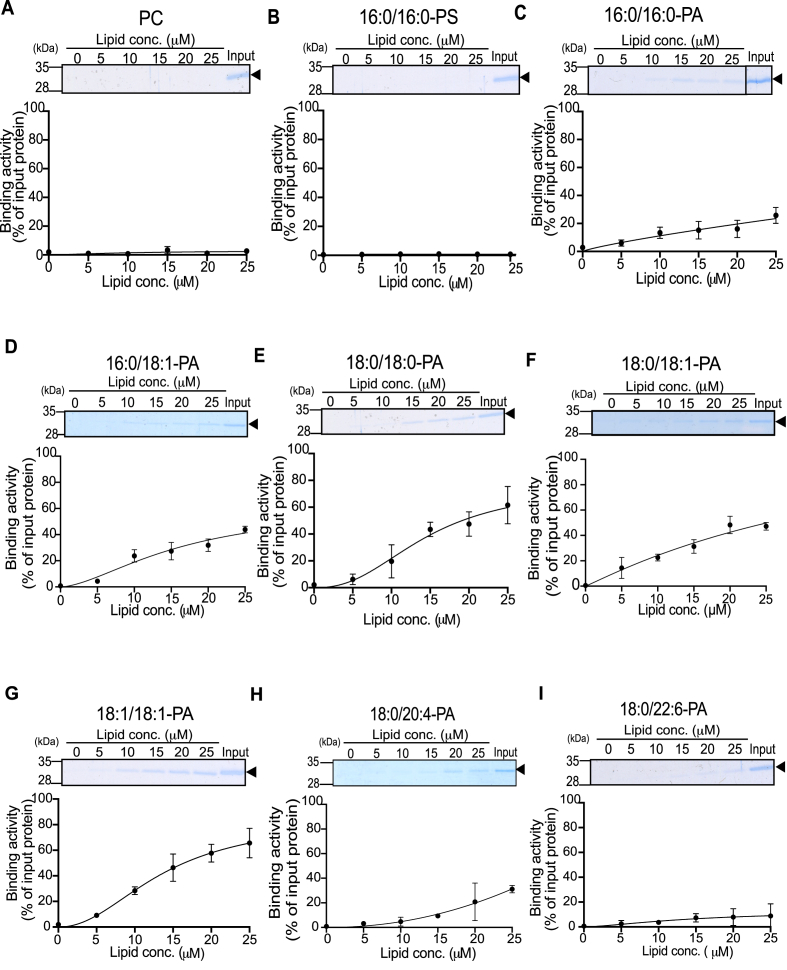
Binding affinity of 6 × His-PGAM1 to different PA species (A–F) Purified 6 × His-PGAM1 (0.2 μM) was incubated with increasing concentrations (0–25 μM) of liposomes containing PC (A), 16:0/16:0-PS (B), 16:0/16:0-PA (C), 16:0/18:1-PA (D), 18:0/18:0-PA (E), 18:0/18:1-PA (F), 18:1/18:1-PA (G), 18:0/20:4-PA (H), and 18:0/22:6-PA (I). After ultracentrifugation, samples were analyzed by SDS–PAGE (15 % acrylamide) and stained with CBB. The position of 6 × His-PGAM1 (approximately 32 kDa) is indicated by a black arrowhead. The amount of protein in the precipitate was quantified by densitometry using the ImageJ software. Binding activity was calculated as the percentage of the precipitate band intensity compared to the total band intensity (*input*). Data are presented as the mean ± SD from three independent experiments. The dissociation constant *K*_d_ was determined using GraphPad Prism 8 (one-phase exponential decay model).

Next, we determined the affinity of PGAM1 for various PA species including 16:0/16:0-, 16:0/18:1-, 18:0/18:0-, 18:0/18:1-, 18:1/18:1-, 18:0/20:4-, and 18:0/22:6-PA by measuring binding activity at concentrations ranging from 0 to 25 μM. Liposome-bound PGAM1 increased in a concentration-dependent manner in all PA species examined (Fig. 3C–I). In contrast, PGAM1 showed negligible binding to liposomes containing 25 μM PC (Fig. 3A) or 16:0/16:0-PS (Fig. 3B). In 15 μM PA-containing liposomes, the percentage of bound PGAM1 varied among different PA species: 16 % (Figs. 3C), 27 % (Fig. 3D), 42 % (Figs. 3E), 32 % (Fig. 3F), 48 % (Figs. 3G), 9 % (Fig. 3H) and 6 % (Fig. 3I) for 16:0/16:0-, 16:0/18:1-, 18:0/18:0-, 18:0/18:1-, 18:1/18:1-, 18:0/20:4- and 18:0/22:6-PA-containing liposomes, respectively. These results indicate that PGAM1 strongly binds to 16:0/16:0-, 16:0/18:1-, 18:0/18:0-, 18:0/18:1-, and 18:1/18:1-PA (≥15 % binding at 15 μM PA), but not to 18:0/20:4- or 18:0/22:6-PA (Fig. 3). The dissociation constants (*K*_d_) of PGAM1 for 18:0/18:0- and 18:1/18:1-PA were determined to be 14.6 and 13.7 μM, respectively (Fig. 3E and G). The *K*_d_ values of PGAM1 for 16:0/16:0-, 16:0/18:1-, 18:0/18:1-, 18:0/20:4- and 18:0/22:6-PA exceeded 25 μM. These findings suggest that PGAM1 has a higher affinity for 18:0/18:0- and 18:1/18:1-PA than for 16:0/16:0-, 16:0/18:1-, and 18:0/18:1-PA (Table 1).

**Table 1:** Summary of the affinity of PA species for PGAM1 (Fig. 3) and their effects on conformational changes (Fig. 4) and activity (Fig. 5) of PGAM1.

	Affinity^a^	Conformational change^b^	Activity (activation)^c^
PC	–	–	–
16:0/16:0-PS	–	–	–
16:0/16:0-PA	++	++	++
16:0/18:1-PA	++	++	++
18:0/18:0-PA	+++	+	+
18:0/18:1-PA	++	++	+
18:1/18:1-PA	+++	++	±
18:0/20:4-PA	+	+	±
18:0/22:6-PA	+	–	–

a +++: ≥40 % binding; ++: ≥15 % binding; +: ≥5 % binding; –: <5 % binding to liposomes containing 15 μM PA species (see Fig. 3).

b ++: α-helix ≤20 % (compared to mock, the ratio is 0.7) and β-sheet ≥26 % (compared to mock, the ratio is 1.3); +: α-helix ≤23 % (compared to mock, the ratio is 0.8) or β-sheet ≥24 % (compared to mock, the ratio is 1.2); –: α-helix >23 % (compared to Mock, the ratio is 0.8) or β-sheet <24 % (compared to mock, the ratio is 1.2) (see Fig. 4).

c ++: ≥130 % (compared to mock); +: ≥115 % (compared to mock); ±: ≥105 % (compared to mock); –: <105 % (compared to mock) (see Fig. 5).

### 3.4 16:0/16:0-PA, 16:0/18:1-PA, 18:0/18:1-PA and 18:1/18:1-PA molecular species alter PGAM1 secondary conformation

To investigate whether the PA molecular species affected PGAM1 conformation (secondary structures), we conducted CD spectroscopy. PGAM1 protein (10 μM) was incubated with liposomes containing PC, PS, or various PA species (16:0/16:0-, 16:0/18:1-, 18:0/18:0-, 18:0/18:1-, 18:1/18:1-, 18:0/20:4-, and 18:0/22:6-PA). CD spectra were measured at wavelengths of 190–250 nm (Fig. 4A). Notably, liposomes containing 16:0/16:0-, 16:0/18:1-, 18:0/18:1-, and 18:1/18:1-PA showed significantly reduced negative maxima at 208 and 222 nm (Fig. 4A), which are characteristic for α‐helices [71]. 18:0/18:0- and 18:0/20:4-PA moderately decreased these maxima (Fig. 4A), whereas 18:0/22:6-PA, PC, and 16:0/16:0-PS showed no effect (Fig. 4A).

**Fig. 4:**
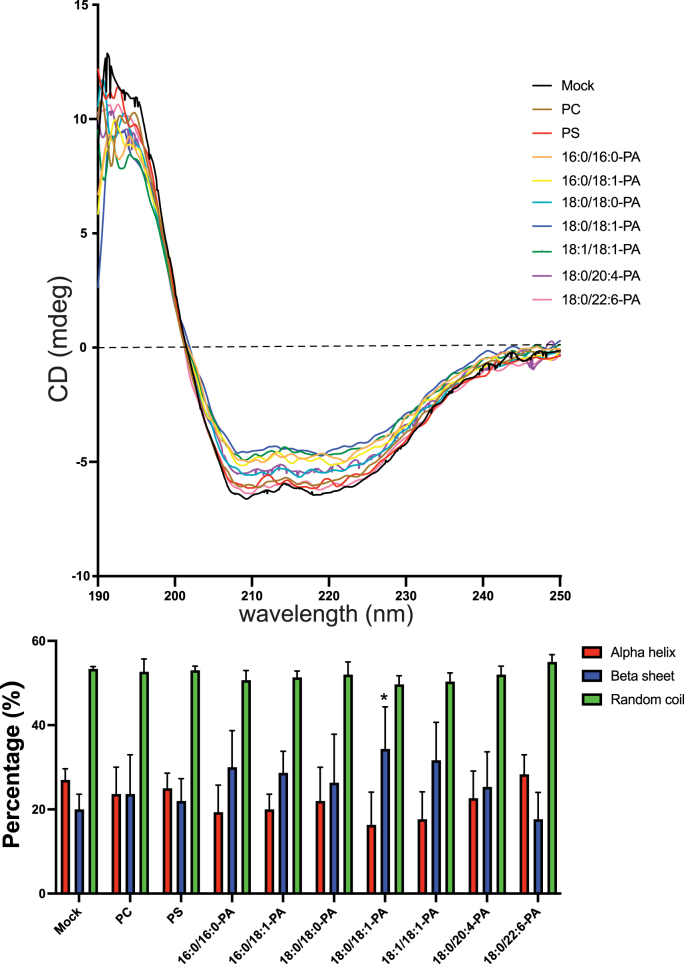
Effect of PA species on the secondary conformation of PGAM1 CD spectra of PGAM1 (16 μM) were measured in 20 mM Tris-HCl buffer (pH 7.4, 1 mM dithiothreitol) between 190 and 250 nm using a Jasco J-805 spectrometer. (A) Spectra were recorded for 6 × His-PGAM1 alone and with PC, 16:0/16:0-PS, 16:0/16:0-PA, 16:0/18:1-PA, 18:0/18:0-PA, 18:0/18:1-PA, 18:1/18:1-PA, 18:0/20:4-PA and 18:0/22:6-PA liposomes (PS and PA: 12.5 μM) added to the protein under the same conditions described above. (B) Secondary structure composition estimated from CD spectra data using the K2D algorithm via the DichroWeb interface. Data are presented as the mean ± SD from three independent experiments. ∗P < 0.05, one-way ANOVA followed by Dunnett's post-hoc test versus PGAM1 alone (mock).

Secondary structural analysis using the K2D algorithm [68] revealed that liposomes containing 16:0/16:0-, 16:0/18:1-, 18:0/18:1-, and 18:1/18:1-PA, but not 18:0/18:0-, 18:0/20:4-, or 18:0/22:6-PA, reduced the α‐helix content (19 %, 19 %, 16 % and 17 %, respectively) compared with PGAM1 alone (27 % α‐helix content) (Fig. 4B). Simultaneously, the β-sheet content (34 %) significantly increased in the presence of 18:0/18:1-PA compared with PGAM1 alone (20 % β-sheet content). In addition, liposomes with 16:0/16:0-, 16:0/18:1-, and 18:1/18:1-PA tended to increase the β-strand content (30 %, 29 % and 31 %, respectively) of PGAM1. These results strongly suggest that 16:0/16:0-, 16:0/18:1-, 18:0/18:1-, and 18:1/18:1-PA selectively induce the conformational changes in PGAM1, decreasing the α‐helical content and increasing the β-strand content (Table 1).

### 3.5 16:0/16:0- and 16:0/18:1-PA significantly enhance PGAM1 activity

**Fig. 5:**
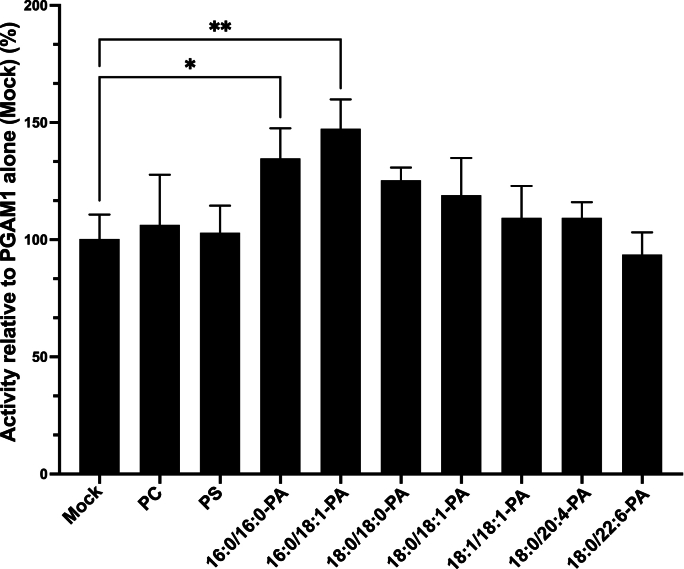
Effect of PA species on PGAM1 activity Purified 6 × His-PGAM1 (0.1 mg/mL) was incubated with PC, 16:0/16:0-PS, 16:0/16:0-PA, 16:0/18:1-PA, 18:1/18:1-PA, 18:0/18:1-PA, 18:0/18:0-PA, 18:0/20:4-PA or 18:0/22:6-PA liposomes (PA: 12.5 μM) in the PGAM assay mixture (100 μL) containing 50 mM HEPES (pH 7.5), 10 mM MgCl_2_, 1 mM ADP, 0.5 units/mL enolase, 0.5 units/mL recombinant pyruvate kinase M1, and 1 mM 3-phosphoglycerate at 25 °C. After incubation for 10 min, ATP production was measured. Data are presented as the mean ± SD from three independent experiments. The activity of PGAM1 alone (mock) was set to 100. ∗P < 0.05, ∗∗P < 0.01, one-way ANOVA followed by Dunnett's post-hoc test versus PGAM1 alone (mock).

We investigated the effects of liposomes containing various PA species on PGAM1 enzymatic activities. Notably, PGAM1 activity was significantly enhanced (more than 30 % increase) by 16:0/16:0- and 16:0/18:1-PA-containing liposomes (Fig. 5 and Table 1). 18:0/18:0- and 18:0/18:1-PA-containing liposomes showed a tendency to increase PGAM activity by ≥ 15 % (Fig. 5 and Table 1). In contrast, 18:1/18:1-, 18:0/20:4-, and 18:0/22:6-PA-containing liposomes, as well as control liposomes (PC and PS), had minimal effects on PGAM activity (≤15 % increase) (Fig. 5). These findings, summarized in Table 1, demonstrate that 16:0/16:0- and 16:0/18:1-PA strongly bind to PGAM1 (Fig. 3), induce conformational changes (Fig. 4), and significantly activate the enzyme (Fig. 5).

### 3.6 PGAM1 selectively interacts with DGKδ and ζ

**Fig. 6:**
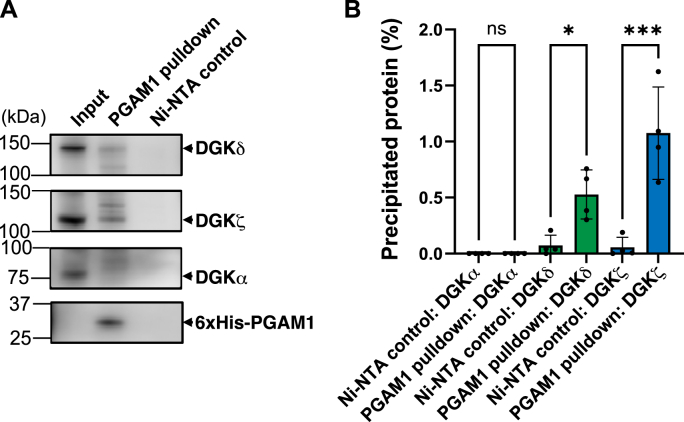
Interaction of PGAM1 with DGKα, δ and ζ **(A)** Western blot analysis of pull-down samples using purified 6 × His-tagged PGAM1 immobilized on Ni-NTA beads and HEK293T lysates endogenously expressing DGKα, δ, and ζ. HEK293T lysates were incubated with PGAM1-bound beads, and eluates were probed with antibodies against DGKα, DGKδ, DGKζ, and 6 × His-tag. Ni-NTA beads without PGAM1 were used as controls. The input lane represents 1.5 % of the total cell lysate. Arrowheads indicate the positions of the DGK isozyme or PGAM1 bands detected using each antibody. The positions of DGKδ (approximately 140 kDa), DGKζ (approximately 110 kDa), DGKα (approximately 80 kDa), and 6 × His-PGAM1 (approximately 32 kDa) are indicated by black arrowheads. **(B)** Quantification of co-sedimented DGK isoforms normalized to input levels (mean ± SD, n = 4). Statistical analysis was performed using one-way ANOVA with Tukey's post-hoc multiple comparisons. ∗P < 0.05, ∗∗∗P < 0.005.

Previous studies have shown that DGKα preferentially produces SFA- and/or MUFA-containing PAs including 16:0/16:0-PA in AKI melanoma cells [44]. DGKδ selectively produces SFA- and/or MUFA-containing PAs, such as 16:0/16:0- and 16:0/18:1-PAs, in myoblasts [52]. DGKζ selectively generates SFA-containing PAs, including 16:0/16:0-PA, in neuronal cells [53]. Therefore, we next investigated the potential interactions between PGAM1 and DGK isoforms. Purified 6 × His-PGAM1 was incubated with HEK293 cell lysates expressing endogenous DGKα, δ, and ζ, and a pull-down assay was performed using Ni-NTA beads. As shown in Fig. 6, DGKδ (approximately 140 kDa) was co-sedimented with PGAM1-bound Ni-NTA beads, but not with Ni-NTA beads alone. DGKζ (approximately 110 kDa) was co-precipitated with PGAM1 (Fig. 6). However, DGKα (approximately 80 kDa) was not pulled down by PGAM1 (Fig. 6). These findings strongly suggest that PGAM1 selectively interacts with DGKδ and ζ.

## 4 Discussion

In this study, we have identified PGAM1 as a new PA-binding protein, thereby adding to the growing list of known PA-binding proteins [[1], [2], [3], [4], [5]]. Moreover, we showed that PGAM1 is a selective and unique PA-binding protein. PGAM1, which plays a crucial role in aerobic glycolysis, is a physiologically and pathologically important enzyme and a promising therapeutic target for cancer [[58], [59], [60]], type 2 diabetes [61], and senescence [62,63]. Given its significance, PGAM1 activity must be tightly regulated. Several factors regulate PGAM1 activity. The cofactor 2,3-bisphosphoglycerate enhances PGAM1 activity by phosphorylating the mutase at H11 in the active site [72]. Complexes formed by Nm23-H1 and glyceraldehyde-3-phosphate dehydrogenase affect PGAM1 activity [73]. p53, a tumor suppressor gene product, reduces PGAM1 levels [[74], [75], [76]]. PGAM1 is regulated by hypoxia-inducible factors and plays an important role in the Warburg effect [58]. The PI 3-kinase/protein kinase B (Akt)/mammalian target of rapamycin (mTOR) pathway positively regulated PGAM1 [77]. Sirtuin 1 and 2, which are NAD^+^-dependent protein deacetylases and are involved in senescence, regulate PGAM1 activity [58,60,[78], [79], [80]]. Our study added lipid molecules, specifically 16:0/16:0- and 16:0/18:1-PA, as PGAM1 activators, expanding the list of regulators beyond phosphorylation, protein-protein interactions and nucleotides.

In recent years, numerous PA-binding proteins have been identified [2,3]. However, many of these proteins also exhibit strong or moderate binding to other acidic phospholipids such as PS, PG, PI, and/or phosphoinositides [2,3]. In contrast, PGAM1 demonstrates high selectivity for PA, interacting strongly with it while only weakly binding to PI-4-P, PS, and CL, and showing no detectable association with other acidic lipids such as PI-4,5-P_2_, PI-3,4,5-P_3_, PI, and PG (Fig. 2). Previously reported PA-binding proteins lack remarkable selectivity for specific PA species [2,3]. For instance, trigalactosyldiacylglycerol 2 [81], protein phosphatase-1 [82], sporulation-specific protein 20 [14], cAMP phosphodiesterase 4A1 [14], and OverProducer of Inositol 1 [14] are associated with a broad range of PA species. However, our recent studies revealed more specific interactions: SFA/MUFA-PA species are associated with and activate CKM [21,23]. 16:0/16:0-PA binds to PI 4,5-bisphosphate-specific PLCβ1 and regulates its subcellular localization (plasma membrane), followed by neurite outgrowth attenuation [18]. 18:1/18:1-PA interacts with α-synuclein and enhances its aggregation [19]. PUFA-containing PA species bind to and moderately inhibit l-lactate dehydrogenase A [23]. A PUFA-containing PA, 18:0/22:6-PA, is associated with Praja-1, a serotonin transporter-selective E3 ubiquitin-protein ligase, and activates it [22]. 18:0/22:6-PA interacts with synaptojanin-1 and enhances its D4-phosphatase activity for PI-4,5-P_2_ [24]. 18:0/22:6-PA is associated with the clathrin coat assembly protein AP180 and inhibits its binding to clathrin [25]. This study added PGAM1 to the list of SFA/MUFA-PA-binding proteins. PGAM1 exhibited remarkable selectivity for SFA/MUFA-PA species and was activated by these species (Fig. 2, Fig. 3, Fig. 5 and Table 1). These findings suggest that individual PA species have distinct physiological functions mediated through their specific binding proteins.

PGAM1 exhibited significant binding affinity for SFA- and/or MUFA-containing PAs, including 16:0/16:0-, 16:0/18:1-, 18:0/18:0-, 18:0/18:1- and 18:1/18:1-PA (≥15 % binding to 15 μM PA-species-containing liposomes), but not to PUFA-containing PAs, 18:0/20:4- and 18:0/22:6-PA (Fig. 3 and Table 1). Among SFA- and/or MUFA-containing PAs, liposomes containing 18:0/18:0-, 18:0/18:1-, and 18:1/18:1-PA sedimented with 42 %, 32 %, and 48 % of PGAM1, respectively (Fig. 3 and Table 1). These findings suggest that SFAs and MUFAs at the *sn*-1 and *sn*-2 positions are critical for association with PGAM1, whereas PUFAs at the *sn*-2 position are unfavorable for PGAM1 binding. The *K*_d_ values of PGAM1 for 18:1/18:1-PA and 18:0/18:0-PA were 14.6 and 13.7 μM, respectively (Fig. 3). These values are comparable to those reported for other PA-binding proteins, including HSP27 (13.3 μM) [54], CKM (2.0 μM) [21], l-lactate dehydrogenase A (3.8 μM) [23], synaptojanin-1 (0.5 μM) [24], α-synuclein (6.6 μM) [20], neurofibromatosis type-1 (12.0 μM) [83], PDE4A1 (6.8 μM) [14], Opi1p (4.5 μM) [14], and sporulation-specific protein 20 (2.0 μM) [14].

CD secondary structure analysis revealed that liposomes containing 16:0/16:0-, 16:0/18:1-, 18:0/18:1- and 18:1/18:1-PA, but not 18:0/18:0-, 18:0/20:4- or 18:0/22:6-PA-containing liposomes, tended to decrease the α‐helix content of PGAM1 compared to PGAM1 alone (27 % α‐helix content) (Fig. 4A and B and Table 1). Simultaneously, these PA species exhibited a tendency to increase in the β-strand content of PGAM1 (Fig. 4A and B and Table 1). Our previous studies have shown that PUFA-containing PA species, such as 18:0/20:4- and 18:0/22:6-PA, which strongly bind to l-lactate dehydrogenase A, selectively induce its conformational changes, decreasing α‐helical content and increasing unordered structure (random coil) content, while moderately inactivating the enzyme [23]. Additionally, we previously reported that SFA/MUFA-PA species strongly bound to Ref. [21] and activated CKM [23] without altering its secondary structures [23]. These findings indicate that diverse PA molecular species affect PA-binding proteins differently.

Regarding PA-binding affinity, PGAM1 exhibits strong binding to 16:0/16:0-, 16:0/18:1-, 18:0/18:0-, 18:0/18:1- and 18:1/18:1-PA, but not to PUFAs (18:0/20:4- and 18:0/22:6-PA) (Fig. 3 and Table 1). Moreover, PGAM1 shows higher affinity for 18:0/18:0- and 18:1/18:1-PA compared to 16:0/16:0-, 16:0/18:1- and 18:0/18:1-PA (Fig. 3 and Table 1). These findings suggest that PGAM1 prefers 18:0 and 18:1, and to a lesser extent 16:0, at the *sn*-1 position, while disfavoring PUFA at the *sn*-2 position. PGAM1 conformation changes are preferentially induced by SFAs and MUFAs at both the *sn*-1 and *sn*-2 positions (Fig. 4 and Table 1). For PGAM1 activation, 16:0 is more effective than 18:0 and 18:1 at the *sn*-1 position, while the *sn*-2 position should be occupied by SFAs or MUFAs, but not PUFAs (Fig. 5 and Table 1). Collectively, 16:0/16:0- and 16:0/18:1-PA demonstrate relatively high affinity for PGAM1, induce strong changes in its secondary structure, and substantially activate the enzyme. However, because the PA-binding site in PGAM1 is unclear, the effects of fatty acids on the secondary structure and activity of PGAM1 are not well understood. Therefore, further research is needed on this point. In melanoma cells, the PA species content follows the order: 16:0/18:1-PA > 16:0/16:0-PA > 18:0/18:1-PA ≈ 18:1/18:1-PA > 18:0/20:4-PA > 18:0/22:6-PA ≫ 18:0/18:0-PA [44]. This distribution suggests that 16:0/18:1-PA and 16:0/16:0-PA may primarily function as PGAM1 activators in cellular environments.

The specific PA-binding site in PGAM1 remains unidentified. However, PGAM1 contains three Lys/Arg clusters (more than three residues, Lys/Arg content ≥40 %) at aa 61–65 (KRAIR), aa 222–228 (KNLKPIK), and aa 251–254 (KAKK). These three Lys/Arg clusters may potentially serve as PA-binding sites.

We next sought to identify the enzymes upstream of PGAM1 that produce 16:0/16:0- and 16:0/18:1-PA. DGKα has been reported to generate 16:0/16:0-, 16:0/18:0-, and 16:0/16:1-PA in melanoma cells [44] and 14:1/16:1-, 14:0/16:1-, 14:0/16:0-, 16:1/16:2-, 16:1/16:1-, 16:0/16:1-, 16:0/16:0-, 16:0/18:1-, and 16:0/18:0-PA in T cells [84]. Additionally, DGKδ selectively produces 14:0/16:0-, 16:0/18:0-, 16:0/16:0-, and 16:0/18:1-PA in high glucose-stimulated C2C12 myoblasts [52]. DGKζ also selectively produces 16:0/16:0-, 14:0/16:0-, and 16:0/18:0-PA in Neuro-2a neuronal cells during differentiation [53]. However, these DGK isozymes do not exhibit DG species selectivity *in vitro* [2,85,86], suggesting that they utilize the SFA-/MUFA-containing DG species available in cells. Our recent studies have identified several enzymes that supply DG upstream of DGK. Sphingomyelin synthase (SMS)-related protein (SMSr) exhibits PLC activities for PI and PC, as well as phosphatase activity for PA [87]. Moreover, SMS1 [88] and SMS2 [89] function as PC-PLC. Furthermore, PHOSPHO1 demonstrates PC- and PE-PLC activities [90]. SMSr [87], SMS1 [88], SMS2 [89], and PHOSPHO1 [90] show a preference for SFA-/MUFA-containing phospholipid species *in vitro* and in cells, preferentially producing SFA/MUFA-containing DG species [[87], [88], [89], [90]]. In addition, DGKδ interacts with SMSr [91] and PHOSPHO1 [90]. DGKζ is associated with SMSr and SMS1 [92]. Although DGKα was not directly associated with SMSr, SMS1, SMS2, or PHOSPHO1, DGKα can still use SFA- and/or MUFA-containing DG species supplied by these DG-generating enzymes because of lateral diffusion of lipids. Therefore, DGKδ, ζ, and α may primarily generate SFA- and/or MUFA-containing PA species using SFA- and/or MUFA-containing DG species supplied by SMSs and PHOSPHO1. We also demonstrated the interaction of PGAM1 with DGKδ and ζ (Fig. 6). This suggests the possibility of pathways involving SMSr/PHOSPHO1–DGKδ–PGAM1 and SMSr/SMS1–DGKζ–PGAM1.

PLD also produces PA [93,94]. However, it is unlikely that PLD primarily generates SFA- and/or MUFA-containing PA species, as it indiscriminately hydrolyzes PC, regardless of the fatty acid composition of PC species, not only SFA- and/or MUFA-containing species but also PUFA-containing species [95].

PGAM1 was strongly implicated in the functional association with DGKδ and ζ (Fig. 6). DGKδ deficiency impairs energy metabolism while exacerbating type 2 diabetes [47]. Consequently, SFA/MUFA-PAs generated by DGKδ may play a crucial role in skeletal muscle energy metabolism through PGAM1 activation. DGKζ generates SFA/MUFA-containing PAs, and up-regulates neurite outgrowth during the initial/early stage of neuroblastoma cell differentiation [53]. In neuronal cells, PA regulates development and increases during differentiation [96]. Energy metabolism, including glycolysis, is essential for neuronal energy demands during neuronal development and function [97], and PGAM1 promotes neuroblast differentiation in the dentate gyrus [98]. Thus, SFA/MUFA-containing PAs produced by DGKζ may be important for neurite outgrowth and neuronal differentiation via PGAM1 activation.

In summary, this study demonstrated (Table 1) that SFA- and/or MUFA-containing PAs, specifically 16:0/16:0- and 16:0/18:1-PA, strongly bind to PGAM1 (Fig. 3), induce conformational changes (Fig. 4), and significantly activate it (Fig. 5). Moreover, PGAM1 was associated with DGKδ and ζ (Fig. 6). PGAM1 is an attractive therapeutic target for cancer [[58], [59], [60]], type 2 diabetes [61], and senescence [62]. Therefore, 16:0/16:0- and 16:0/18:1-PA and their analogs can serve as regulatory molecules for these diseases and disorders through the modulation of PGAM1 functions. However, further studies are needed to confirm the functional links between PGAM1, DGKδ/ζ, and SFA/MUFA-PA species *in vivo*.

## CRediT authorship contribution statement

**Kamila Dilimulati:** Writing – review & editing, Writing – original draft, Investigation, Funding acquisition. **Naoto Yachida:** Writing – review & editing, Writing – original draft, Investigation, Conceptualization. **Fumi Hoshino:** Writing – review & editing, Investigation. **Fumio Sakane:** Writing – review & editing, Writing – original draft, Supervision, Project administration, Funding acquisition, Conceptualization.

## Data availability statement

The data that support the findings of this study are available from the corresponding author upon reasonable request.

## Declaration of generative AI and AI-assisted technologies in the writing process

During the preparation of this work including the writing process, the authors did not use any generative AI or AI-assisted technologies.

## Declaration of competing interest

The authors declare no conflicts of interest associated with the content of this article.
