# Supplementary material for: Saturated fatty acid- and/or monounsaturated fatty acid-containing-phosphatidic acids selectively interact with and activate phosphoglycerate mutase 1
Source: Biochem Biophys Rep. 2025 Sep 29;44:102285. doi: 10.1016/j.bbrep.2025.102285 (PMC12513065; doi:10.1016/j.bbrep.2025.102285)
Supplement: Multimedia component 1 [file mmc1.pdf]

A

Accession No.	M. W.	-10lgP	Coverage (%)	#Unique peptides	Protein name
P18669	28804	173.22	36	10	Phosphoglycerate mutase 1 (PGAM1)

B

Accession No.	M. W.	SCORE	emPAI	Protein name
O70250	28980	130	0.72	Phosphoglycerate mutase 2 (PGAM2)

Suppl. Fig. 1. Identification of 16:0/16:0-PA-binding proteins in human melanoma cells (A) and mouse skeletal muscle (B).
